# Factors Associated with Loss to Follow-up among Cervical Cancer Patients in Rwanda

**DOI:** 10.5334/aogh.2722

**Published:** 2020-09-14

**Authors:** Placide Habinshuti, Marc Hagenimana, Cam Nguyen, Paul H. Park, Tharcisse Mpunga, Lawrence N. Shulman, Alexandra Fehr, Gilbert Rukundo, Jean Bosco Bigirimana, Stephanie Teeple, Catherine Kigonya, Gilles Francois Ndayisaba, Francois Uwinkindi, Thomas Randall, Ann C. Miller

**Affiliations:** 1 Partners in Health/Inshuti Mu Buzima, RW; 2 Rwanda Biomedical Center/Non-Communicable Diseases division, RW; 3 Athena Institute of Vrije Universiteit, NL; 4 Partners in Health/Boston, US; 5 Harvard Medical School, US; 6 Brigham and Women’s Hospital, US; 7 Ministry of Health/Butaro Hospital, RW; 8 University of Pennsylvania, US; 9 Massachusetts General Hospital, US

## Abstract

**Background:**

Cervical cancer is among the most common cancers affecting women globally. Where treatment is available in low- and middle-income countries, many women become lost to follow-up (LTFU) at various points of care.

**Objective:**

This study assessed predictors of LTFU among cervical cancer patients in rural Rwanda.

**Methods:**

We conducted a retrospective study of cervical cancer patients enrolled at Butaro Cancer Center of Excellence (BCCOE) between 2012 and 2017 who were either alive and in care or LTFU at 12 months after enrollment. Patients are considered early LTFU if they did not return to clinic after the first visit and late LTFU if they did not return to clinic after the second visit. We conducted two multivariable logistic regressions to determine predictors of early and late LTFU.

**Findings:**

Of 652 patients in the program, 312 women met inclusion criteria, of whom 47 (15.1%) were early LTFU, 78 (25.0%) were late LTFU and 187 (59.9%) were alive and in care. In adjusted analyses, patients with no documented disease stage at presentation were more likely to be early LTFU vs. patients with stage 1 and 2 when controlling for other factors (aOR: 14.93, 95% CI 6.12–36.43). Patients who travel long distances (aOR: 2.25, 95% CI 1.11, 4.53), with palliative care as type of treatment received (aOR: 6.65, CI 2.28, 19.40) and patients with missing treatment (aOR: 7.99, CI 3.56, 17.97) were more likely to be late LTFU when controlling for other factors. Patients with ECOG status of 2 and higher were less likely to be late LTFU (aOR: 0.26, 95% CI 0.08, 0.85).

**Conclusion:**

Different factors were associated with early and later LTFU. Enhanced patient education, mechanisms to facilitate diagnosis at early stages of disease, and strategies that improve patient tracking and follow-up may reduce LTFU and improve patient retention.

## Introduction

Cervical cancer is among the most common cancers and is the fourth most common cause of cancer death in women worldwide [1]. Women in low- and middle-income countries (LMICs) disproportionately bear the burden of cervical cancer; 85% of cervical cancer morbidity and 88% of cervical cancer mortality occur in this region [2,3,4]. In East Africa, among all types of cancers in women, cervical cancer is the leading cause of morbidity and mortality with 52,633 new cases and 37,017 deaths estimated in 2018 [5]. Without adequate investment in cervical cancer control, these rates are only expected to rise [2].

Treatment for cervical cancer is critical for control and secondary disease prevention in LMICs [2]. However, most LMICs have limited infrastructure and human resource capacity to support surgical screening and subsequent treatment with radiotherapy, evidenced by the lack of trained health personnel and inadequate availability of treatment equipment [2]. Where services are available, the cost of treatment often prohibits access [6,7]. Further, issues such as late presentation at diagnosis, low pre-treatment performance status, which indicates a patient’s ability to tolerate chemotherapy, lack of adherence to treatment or post-treatment follow-up, and low quality of care worsen patient outcomes [2,8,9,10,11].

Among important programmatic and patient-related aspects of cervical cancer treatment is post-treatment follow-up. Women receiving therapy for invasive cancer may experience severe side effects and disease-related complications that require monitoring and intervention from cancer specialists. However, high proportions of loss to follow-up (LTFU) for cervical cancer patients have been reported in LMICs, ranging between 41–69% [10,12]. Several studies have reported that socio-demographic factors such as age, religion, marital status, distance to cancer treatment center, and education level are associated with LTFU from cancer care [8,13,14,15]. However, few of these studies have been conducted in rural settings; therefore little is known about cervical cancer patient LTFU patterns in non-urban contexts.

Rwanda, a predominantly rural East African country, reports similar cervical cancer morbidity and mortality rates as compared to other LMICs. In 2018, an estimated 1,304 women were newly diagnosed with cervical cancer and 921 cervical cancer deaths occurred [5]. Cancer diagnosis, disease staging and surgical treatment are available at five referral hospitals in the country. Of these facilities, the Butaro Cancer Center of Excellence (BCCOE) is the first and, currently, only public cancer treatment center located in a rural setting specializing in diagnostics, chemotherapy and follow-up services for cancer patients and has provided treatment and follow-up services for cervical cancer patients since 2012. This study assessed the rates of and factors associated with LTFU among these patients to help inform policy and practice in Rwanda and other similar settings.

## Methods

### Study setting

BCCOE opened in July 2012 as a joint venture between the Rwandan Ministry of Health, the international non-governmental organization Partners in Health/Inshuti Mu Buzima (PIH/IMB), and other partners. BCCOE is located within the public district hospital in rural Burera District, approximately 93 km from the capital city of Kigali and accessible by road and public transportation. The hospital has a capacity of 167 beds. Since the opening of the cancer center, over 10,000 patients from Rwanda and surrounding countries have been enrolled in the cancer program. BCCOE provides histopathology-based diagnosis, basic imaging (X-ray, ultrasound), chemotherapy, limited targeted therapy and surgical procedures, palliative care and socioeconomic support. Specialized surgeries and more advanced imaging (CT and MRI) are performed at teaching hospitals in the capital city. Patients needing radiotherapy are referred to tertiary hospitals in Uganda and Kenya, the cost of which is subsidized by PIH/IMB for a selected number of patients with the best estimates of prognosis and benefit from radiotherapy. Care at BCCOE is delivered by general physicians, internists, and pediatricians using a task-shifting and twinning model supported by oncologists from US cancer centers, and nationally endorsed protocols adapted to available resources. After breast cancer, cervical cancer is the second most common diagnosis at BCCOE.

### Study design and population

**Figure 1:**
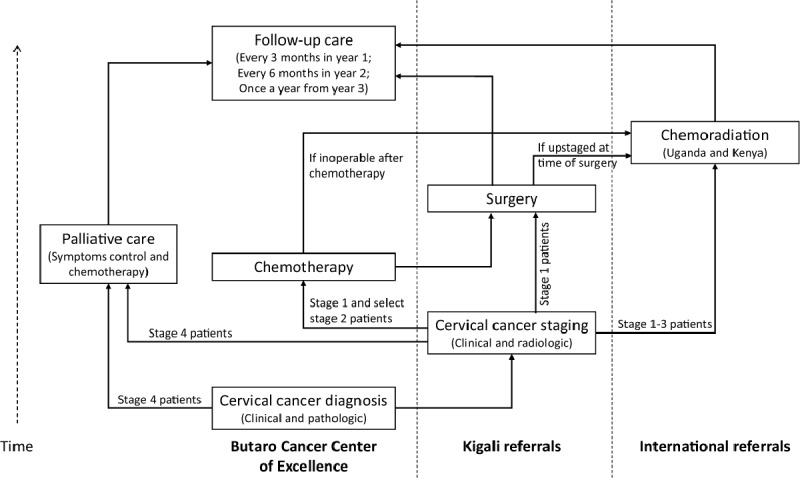
Treatment flowchart for cervical cancer patients at Butaro Cancer Centre of Excellence (BCCOE). For curative treatment, pre-operative chemotherapy is provided at BCCOE and surgery at referral hospitals in Kigali while chemoradiation requires referrals to facilities outside of Rwanda. Palliative care can include chemotherapy and radiotherapy; however, only symptom control and chemotherapy are available for palliative care at BCCOE.

We conducted a retrospective cohort study of all cervical cancer patients who presented for treatment at BCCOE between 1 July 2012 and 30 June 2017. Patients follow a specific pathway, depending on disease severity and type of treatment required. Figure 1 serves to visually demonstrate the cervical cancer treatment pathway in Rwanda.

After a pathologically-confirmed diagnosis of cervical cancer, and if not yet staged at presentation, women with non-metastatic disease are referred to referral hospitals in Kigali for clinical and radiologic disease staging according to the International Federation of Gynecology and Obstetrics (FIGO) system. Upon staging, while chemoradiation is the preferred treatment for all non-metastatic patients, where radiotherapy is limited, like in Rwanda, one of three pathways is followed: 1) for early stage disease, patients are referred to referral hospitals in Kigali for surgical resection prior to or after completion of neo-adjuvant chemotherapy at BCCOE. Some patients with early stage disease may be further referred for concurrent chemoradiation if they are found inoperable after chemotherapy or are upstaged at the time of surgery. 2) For locally advanced stage disease, patients are referred to hospitals in Uganda and Kenya for chemoradiation. 3) For late stage or metastatic disease, patients receive palliative support either at their local district hospital or at BCCOE. Palliative care services focus on treating the symptoms and stress related to cancer diseases. These include pain relief, and spiritual, emotional, psychosocial, socio-economic and, in some settings, nutritional support. After treatment, all patients are followed at BCCOE and evaluated for disease recurrence or progression. Patients are asked to return every three months for the first year, every six months for the second year and once per year thereafter. Further details about cervical cancer treatment at BCCOE can be found in Park et al., 2018 [16].

### Data collection and analysis

We extracted patient demographic data, including: age, residence (Northern Province [site of BCCOE] vs. all others), type of health insurance, and referring health facility. We gathered clinical characteristics including: history of tobacco use and alcohol consumption, use of traditional medicine, HIV status, duration of chief complaint prior to diagnosis (less than 6 months, 6–11 months, 12 months or more), non-communicable disease (NCD) comorbidities, including epilepsy, hypertension, diabetes, asthma or renal disease, and cancer stage at presentation (dichotomized as early stage [stage 1 or 2] vs. late stage [stage 3 or 4]).

We also collected physical function test results (Eastern Cooperative Oncology Group [ECOG]) and dichotomized responses as 0 (score 0 or 1: none to minor to moderate activity restrictions, up and around more than 50% of the time) vs. 1 (score 2 or higher: significant active restriction or disability, confined to bed or chair more than 50% of the time) [17]. Treatment type received was categorized as curative chemoradiation, curative chemotherapy or palliative treatment.

We also collected information on visit dates from the electronic medical record system and performed manual chart reviews to ensure data completeness and accuracy. We included missing data as a category in most variables as an important indicator of retention in program and quality of programmatic record-keeping.

**Figure 2:**
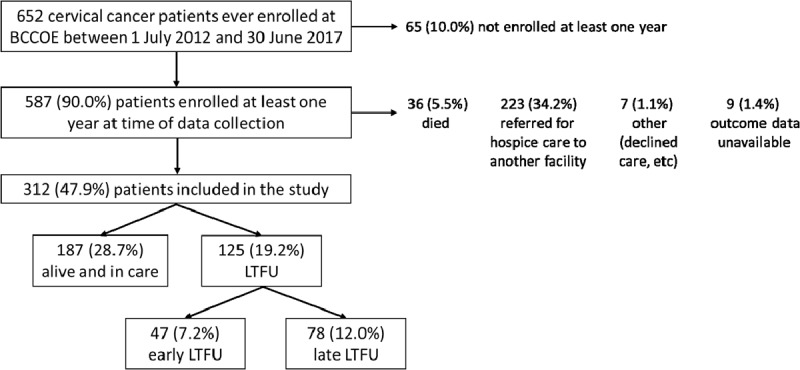
Flowchart of patients selected based on inclusion and exclusion criteria. Patients who had been enrolled at least one year at the time of data collection and were either alive and in care or lost-to follow-up (LTFU) one year after enrollment were included in the study. All percentages were calculated on the 652 patients enrolled at Butaro Cancer Center of Excellence (BCCOE) between 1 July 2012 and 30 June 2017.

Our analysis included only patients who, at the time of data collection, had been enrolled at BCCOE for at least one year and were in care or lost to follow up (LTFU) at one year after enrollment. Patients who transferred out of the program to another facility, were referred elsewhere for hospice care, refused care, or died before one year of enrollment were not included in the analysis (Figure 2).

The primary outcome was LTFU versus in care at one year after enrollment at BCCOE. Patients were considered LTFU if, despite phone calls from nurses, they did not return to care within six months after a missed visit. For patients who did not have a recorded next scheduled visit, LTFU was defined as no completed visit within one year after the last completed visit. “Early LTFU” was defined as completing no additional visits after the first visit to the oncology program; “late LTFU” was defined as no completed visit at any point after completing at least two visits.

We conducted all analyses using STATA version 15 (Stata, College Station, TX), and conducted separate analyses for factors associated with early and late LTFU. We described patient characteristics and outcomes using frequencies and percentages grouped by early LTFU, late LTFU and in care. We assessed associations of treatment details, socio-demographic and clinical characteristics with early and late LTFU at one year using Chi-square and Fisher’s exact tests, reporting p-values. All variables in the bivariate analysis that were significant at an alpha level of <0.2 were considered for multivariable analysis using logistic regression. We conducted multivariable analyses separately for early and late LTFU reporting odds ratios (OR), 95% confidence intervals, and p-values, significant at an alpha of 0.05, for unadjusted, fully adjusted and final models, using a backward stepwise method.

### Ethics

This study was reviewed and approved by the Rwanda National Ethics Committee as part of a larger protocol on the delivery of cancer care in Rwanda. The study was given technical approval by Partners in Health/Inshuti Mu Buzima (IMB) Research Committee.

## Results

Among 652 cervical cancer patients registered at BCCOE between July 2012 and June 2017, 65 (10.0%) were not enrolled for at least a year, 36 (5.5%) had died, 223 (34.2%) had been referred for hospice care, 7 (1.1%) had other outcomes, and 9 (1.4%) had no outcome data available (Figure 2). Of the 312 patients who were eligible and included in our study, 47 (15.1%) were considered early LTFU, 78 (25.0%) were late LTFU, and 187 (59.9%) were retained in care. Of the 312 patients included, 194 (62.2%) were aged 50 years and above at the time of diagnosis, 118 (37.8%) were HIV positive, and 233 (74.7%) had stage 1–2 disease, 41 (13.1%) stage 3–4, while the remaining 38 (12.2%) patients had no documented disease stage.

Among the patients who were early LTFU, the largest proportion were from the Northern Province (19, 43.2%) and the majority had no stage documented at presentation (21, 44.7%). Among late LTFU patients, the largest proportion were from Kigali (30, 40.0%) and the majority had stage 1 or 2 disease at presentation (61, 78.2%). Additionally, the majority experienced at least one adverse effect at a follow-up visit post-treatment (20, 58.8%). Among patients who were alive and in care, the largest proportion were from Kigali (76, 40.6%), and the majority also had stage 1 or 2 disease at presentation (153, 81.8%). Additionally, the majority experienced adverse effect at follow-up (121, 80.1%) (Table 1).

**Table 1:** Demographic and clinical characteristics of cervical cancer patients at BCCOE enrolled between July 2012 and June 2017 who were early LTFU, late LTFU or alive and in care. N = 312 unless otherwise specified.

	Early LTFU N = 47		Late LTFU N = 78		Alive and in care N = 187	

	n	%	n	%	n	%

**Age**						
39 or younger	6	12.8	3	3.9	19	10.2
40–49	13	27.7	26	33.3	51	27.3
50–59	13	27.7	26	33.3	66	35.3
60–69	10	21.3	15	19.2	40	21.4
70 or older	5	10.6	8	10.3	11	5.9
**Residence**						
Northern Province	19	40.4	16	20.5	58	31.0
Other	28	59.6	62	79.5	129	69.0
**Province (n = 306)**						
Kigali	12	27.3	30	40.0	76	40.6
Eastern	3	6.8	6	8.0	21	11.2
Northern	19	43.2	16	21.3	58	31.0
Southern	3	6.8	13	17.3	9	4.8
Western	7	15.9	10	13.3	23	12.3
**Type of insurance (n = 301)**						
National health insurance	39	90.7	70	92.1	169	92.9
Other	4	9.3	6	7.9	13	7.1
**Referring health facility (n = 298)**						
No referral	5	11.9	6	7.9	20	11.1
Referral hospital	11	26.2	25	32.9	62	34.4
District hospital	24	57.2	42	55.3	93	51.7
Other	2	4.8	3	4.0	5	2.8
**Smoking history (n = 300)**						
Current	0	0.0	0	0.0	1	0.6
Past	16	35.6	30	39.5	61	34.1
Never	29	64.4	46	60.5	117	65.4
**Drinking history**						
Current	1	2.1	4	5.1	9	4.8
Past	16	34.0	27	34.6	58	31.0
Never	0	0.0	1	1.3	2	1.1
Missing	30	63.8	46	59.0	118	63.1
**Ever used traditional medicine (n = 281)**						
No	26	60.5	42	57.5	100	60.6
Yes	17	39.5	31	42.5	65	39.4
**Duration of chief complaint (n = 279)**						
Less than six months	13	32.5	27	37.0	58	34.9
6–11 months	8	20.0	18	24.7	38	22.9
12 months or more	19	47.5	28	38.4	70	42.2
**ECOG**						
0–1	35	74.5	68	87.2	146	78.1
2+	6	12.8	6	7.7	18	9.6
Missing	6	12.8	4	5.1	23	12.3
**HIV status**						
Positive	11	23.4	29	37.2	78	41.7
Negative	30	63.8	37	47.4	84	44.9
Missing	6	12.8	12	15.4	25	13.4
**Has NCD comorbidities (n = 288)**						
No	38	88.4	66	94.3	165	94.3
Yes	5	11.6	4	5.7	10	5.7
**Cancer stage at presentation**						
Stage 1 or 2	19	40.4	61	78.2	153	81.8
Stage 3 or 4	7	14.9	11	14.1	23	12.3
Unstaged	21	44.7	6	7.7	11	5.9
**Type of treatment received**						
Curative chemotherapy and radiotherapy	0	0.0	28	35.9	121	64.7
Curative chemotherapy only	0	0.0	7	9.0	20	10.7
Curative radiotherapy only	0	0.0	10	12.8	25	13.4
Curative intent, no treatment recorded	12	25.5	16	20.5	10	5.4
Palliative treatment	7	14.9	10	12.8	8	4.3
Missing	28	59.6	7	9.0	3	1.6
**Experienced chemotherapy toxicity (n = 178)**						
No	0	0.0	21	58.3	82	57.8
Yes	0	0.0	15	41.7	60	42.3
**Experienced radiotherapy toxicity (n = 189)**						
No	0	0.0	17	43.6	84	56.0
Yes	0	0.0	22	56.4	66	44.0
**Experienced adverse effect at follow up (n = 186)**						
No	1	2.2	14	41.2	30	19.9
Yes	0	0.0	20	58.8	121	80.1

In the bivariate analysis, HIV status (p-value: 0.048), having at least one NCD comorbidity (p-value: 0.170), and cancer stage at presentation (p-value < 0.001) were statistically associated with early LTFU at an alpha level of <0.2 (Table 2).

**Table 2:** Association between covariates and early LTFU for patients with cervical cancer presenting at BCCOE from July 2012 to June 2017. N = 234 unless otherwise specified. Late LTFU patients were excluded from this analysis.

Variable	Early LTFU		Alive and in care		p-value

	n	%	n	%	

**Age**					
39 or younger	6	24.0	19	76.0	0.668
40–49	13	20.3	51	79.7	
50–59	13	16.5	66	83.5	
60–69	10	20.0	40	80.0	
70 or older	5	31.3	11	68.7	
**Residence**					
Northern Province	19	24.7	58	75.3	0.220
Other	28	17.8	129	82.2	
**Type of insurance (n = 224)**					
National health insurance	39	18.8	169	81.2	0.517
Other insurance or no insurance	4	25.0	12	75.0	
**Referring health facility (n = 222)**					
No referral	5	20.0	20	80.0	0.630
Referral hospital	11	15.1	62	84.9	
District hospital	24	20.5	93	79.5	
Other	2	28.6	5	71.4	
**Smoking history (n = 224)**					
Current	0	0.0	1	100.0	0.890
Past	16	20.8	61	79.2	
Never	29	19.8	117	80.1	
**Drinking history (n = 234)**					
Current	1	10.0	9	90.0	0.897
Past	16	21.6	58	78.4	
Never	0	0.0	2	100.0	
Missing	30	20.3	118	79.7	
**Uses/Used traditional medicine (n = 208)**					
No	26	20.6	100	79.4	>0.999
Yes	17	20.7	65	79.3	
**Duration of chief complaint (n = 206)**					
Less than six months	13	18.3	58	81.7	0.823
6–11 months	8	17.4	38	82.6	
12 months or more	19	21.3	70	78.7	
**ECOG (n = 234)**					
0–1	35	19.3	146	80.7	0.806
2+	6	25.0	18	75.0	
Missing	6	20.7	23	79.3	
**HIV status (n = 234)**					
Negative	30	26.3	84	73.7	0.048
Positive	11	12.4	78	87.6	
Missing	6	19.4	25	80.6	
**Has NCD comorbidities (n = 218)**					
No	38	18.7	165	81.3	0.170
Yes	5	33.3	10	66.7	
**Cancer stage at presentation (n = 234)**					
Stage 1 or 2	19	11.1	153	88.9	<0.001
Stage 3 or 4	7	23.3	23	76.7	
Unstaged	21	65.6	11	34.4	
**Type of treatment received (n = 234)**					
Curative chemotherapy and radiotherapy	0	0.0	146	100.0	<0.001
Curative chemotherapy only	0	0.0	20	100.0	
Palliative treatment	7	46.7	8	53.3	
Missing	40	75.5	13	24.5	

For late LTFU patients, residence outside of Northern Province (p-value: 0.082), ECOG status (p-value: 0.168), and type of treatment received (p-value <0.001) were statistically associated with late LTFU at alpha <0.2 (Table 3).

**Table 3:** Association between different variables and late LTFU for patients with cervical cancer presenting at BCCOE from July 2012 to June 2017. N = 265 unless otherwise specified. Early LTFU patients were excluded from this analysis.

Variable	Late LTFU		Alive and in care		p-value

	n	%	n	%	

**Age**					
39 or younger	3	13.6	19	86.4	0.286
40–49	26	33.8	51	66.2	
50–59	26	28.3	66	71.7	
60–69	15	27.3	40	72.7	
70 or older	8	42.1	11	57.9	
**Residence**					
Northern Province	62	32.5	129	67.5	0.082
Other	16	21.6	58	78.4	
**Type of insurance (n = 235)**					
National health insurance	70	29.3	169	70.7	0.790
Other insurance or no insurance	6	33.3	12	66.7	
**Referring health facility (n = 166)**					
No referral	6	23.1	20	76.9	0.812
Referral hospital	25	28.7	25	71.3	
District hospital	42	31.1	42	68.9	
Other	3	37.5	3	62.5	
**Smoking history (n = 255)**					
Current	0	0.0	1	100.0	0.632
Past	30	33.0	61	67.0	
Never	46	28.2	117	71.8	
**Drinking history**					
Current	4	30.8	9	69.2	0.909
Past	27	31.8	58	68.2	
Never	1	33.3	2	66.7	
Missing	46	28.1	118	71.9	
**Uses/Used traditional medicine (n = 238)**					
No	42	29.58	100	70.4	0.670
Yes	31	32.29	65	67.7	
**Duration of chief complaint (n = 239)**					
Less than six months	27	31.76	58	68.2	0.888
6–11 months	18	32.14	38	67.9	
12 months or more	28	28.57	70	71.4	
**ECOG**					
0–1	68	31.8	146	68.2	0.168
2+	6	25.0	18	75.0	
Missing	4	14.8	23	85.2	
**HIV status**					
Negative	37	30.6	84	69.4	0.772
Positive	29	27.1	78	72.9	
Missing	12	32.4	25	67.6	
**Has NCD comorbidities (n = 245)**					
No	66	28.6	165	71.4	>0.999
Yes	4	28.6	10	71.4	
**Cancer stage at presentation**					
Stage 1 or 2	61	28.5	153	71.5	0.730
Stage 3 or 4	11	32.4	23	67.6	
Unstaged	6	35.3	11	64.7	
**Type of treatment received**					
Curative chemotherapy and radiotherapy	38	20.6	146	79.4	<0.001
Curative chemotherapy only	7	25.9	20	74.1	
Palliative treatment	10	55.6	8	44.4	
Missing	23	63.9	13	36.1	

In the multivariable logistic regression, patients with no documented stage of disease at presentation were more likely to be early LTFU (OR: 14.93, 95% CI 6.12, 36.43) compared to patients who had stage 1 or 2 (Table 4). Similarly, patients with cervical cancer stage 3 or 4 were more likely to be early LTFU (OR: 2.49, 95% CI 0.93, 6.65) as compared to patients with stage 1 or 2. While no other factors were significantly associated with early LTFU, patients who were HIV positive were less likely to be early LTFU (OR: 0.46, 95% CI 0.20, 1.06).

**Table 4:** Multivariable logistic regression for predictors of early LTFU presenting odds ratios, 95% confidence intervals and p-value for patients with cervical cancer presenting at BCCCOE from July 2012 to June 2017.

Variable	Unadjusted bivariate ORs			Final Model		

	OR	95% CI	p-value	OR	95% CI	p-value

**NCD comorbidities**						
No Comorbidities	1					
At least 1 NCD comorbidity	2.17	0.70–6.72	0.180			
**HIV status**						
Negative	1			1		
Positive	0.39	0.18–0.84	0.020	0.46	0.20–1.06	0.071
Missing	0.67	0.25–1.79	0.430	0.51	0.25–1.80	0.430
**Cancer stage at presentation**						
Stage 1 or 2	1			1		
Stage 3 or 4	2.45	0.93–6.47	0.069	2.49	0.93–6.65	0.069
Unstaged	15.37	6.43–36.74	<0.001	14.93	6.12–36.43	<0.001

Among patients classified as late LTFU, those living outside the Northern Province were more likely to be LTFU (OR: 2.25, 95% CI 1.11, 4.53) as compared to those living close to BCCOE (Table 5). Patients missing ECOG score were less likely to be LTFU (OR: 0.26, 95% CI 0.08, 0.85) compared to patients in 0 to 1 as ECOG status. Patients who had palliative treatment at BCCOE (OR: 6.65, 95% CI 2.28, 19.40) and treatment missing (OR: 7.99, 95% CI 2.28, 17.97) were more likely to be LTFU compared to patients who received curative chemotherapy and radiotherapy.

**Table 5:** Multivariable logistic regression for predictors of late LTFU presenting odds ratios, 95% confidence intervals and p-value for patients with cervical cancer presenting at BCCCOE from July 2012 to June 2017.

Variable	Unadjusted ORs			Final Model		

	OR	95% CI	p-value	OR	95% CI	p-value

**Residence**						
Northern Province	1			1		
Other Province	1.74	0.93–3.27	0.080	2.25	1.11–4.53	0.024
**ECOG**						
0–1	1			1		
2+	0.71	0.27–1.88	0.490	0.71	0.25–2.07	0.535
Missing	0.37	0.12–1.12	0.080	0.26	0.08–0.85	0.026
**Type of treatment received**						
Curative chemotherapy and radiotherapy	1			1		
Curative chemotherapy only	1.34	0.53–3.41	0.530	1.15	0.58–3.87	0.408
Palliative treatment	4.8	1.77–13.00	0.002	6.65	2.28–19.40	0.001
Missing	6.79	3.15–14.65	<0.001	7.99	3.56–17.97	<0.001

## Discussion

We assessed factors associated with early and late LTFU among cervical cancer patients seen at a rural cancer center in Rwanda. Among all patients attending BCCOE for cancer care during our study period, the overall LTFU rate was 19%. Even among patients who were referred for curative chemoradiation treatment outside Rwanda, 20% were late LTFU. These proportions are lower than those reported in other LMICs, which can range from 41–69%, demonstrating remarkable retention at this site, despite huge logistical challenges [8,12]. We attribute this result to the fact that BCCOE has a good system of tracking patients who have missed a scheduled appointment and provides financial support, including transportation reimbursement, to the poorest patients to address the barrier of financial accessibility.

In our study, the only factor associated with early LTFU in adjusted analysis was cancer stage at presentation. Patients who had cancer stage 3 or 4 at presentation were more likely to be LTFU after the first visit compared to patients who were in stage 1 or 2, although this difference was only borderline statistically significant (p-value: 0.07). Many studies have investigated the association between late stage disease at presentation and low survival rather than LTFU [11,12,18,19]. A few studies that have assessed stage at presentation and LTFU in South India showed similar associations between advanced stage at presentation and being LTFU [8,10]. Patients arriving into care at a later stage of illness may seek care elsewhere or outside the system if cure within the formal system is deemed unlikely. In our setting, there are few treatment options for late stage disease and the patients in this stage may find it not worth traveling from their homes to BCCOE.

Additionally, this study found that a significantly higher proportion of patients with unknown stage at presentation were more likely to be early LTFU compared to patients whose cancer had been staged as 1 or 2, although these numbers are small and the confidence interval wide. This finding is also similar to Misu et al.’s (2010) finding from India that patients with an unknown stage of cervical cancer had a high likelihood of being LTFU, and consistent with outcomes from a Nigerian study showing that the majority of patients were LTFU prior to staging [8,20]. This may be related to the many travel and logistical challenges associated with the process of staging, so more patients are likely to drop out once they are referred for staging. BCCOE has limited medical imaging facilities and gynecology specialists to provide cancer staging. Thus, patients are typically referred to the capital city (approximately a 2.5-hour drive in a private car, longer by bus) for clinical and radiologic staging, with follow-up in different settings depending on stage. Strengthening BCCOE’s cancer staging capacity may reduce the patient navigation challenges and LTFU. Qualitative research with patients and/or their families may also shed some light upon this issue.

In unadjusted analysis, patients who were HIV positive were less likely to be LTFU after a first visit compared to patients who were HIV negative, although this finding did not retain significance when adjusting for cancer stage at presentation. This non-significant but suggestive association could be explained by the fact that HIV positive patients may already be attending care in an HIV program and are already concerned about their health. Previous studies have also found similar results, although a study conducted in southwestern Nigeria found no significant associations between HIV status and LTFU among cervical cancer patients [15,18,21]. Another study that followed a cohort of HIV positive women with cervical cancer found that patients who had already achieved control for their HIV were more likely to undertake cervical cancer screening [22]. HIV negative patients may have less experience with the health care system, and greater attention to orienting them or accompanying them through care navigation may be of great benefit in retaining these patients.

This study also found significant associations between residence and late LTFU. Patients from outside the Northern Province were more likely to be late LTFU compared to patients who were from within the province. This finding is consistent with other studies which found that patients traveling long distances from the health facility are more likely to be LTFU [8,15,23,24]. At BCCOE, eligible cancer patients with limited financial means are refunded their transport fees, which may contribute to this study’s lower percent of LTFU as compared to other settings. However, it is possible that transport support may not be sufficient to address other barriers patients may face, including travelling time conflicting with other priorities, ability to travel and ability to pay up front for transport. Providing vouchers up front instead of reimbursing costs at a later time may be more effective.

Patients with reduced performance status (ECOG 2+) or whose ECOG was missing were less likely to be LTFU after their second visit compared to patients with ECOG of 0 or 1. Our finding was inconsistent with studies conducted in India that showed patients with worse pre-treatment performance status were at increased risk of LTFU [7,9]. The reasons for high LTFU likelihood in patients with good performance status in our setting should be further analyzed. However, this result might also be a function of incomplete data. A high proportion of patients in our study had missing ECOG, and 75% of these patients had cancer stage 1 or 2 (data not shown), hence, are more likely have an ECOG of 0–1, which should be less likely to be LTFU based on current literature. Although not statistically significant, in general, a lower percent of patients with a cancer stage of 1 or 2 were LTFU. Reinforcement of the importance of assessing and recording this patient information with the clinical staff may be advisable for the program.

Patients who were in palliative care at BCCOE as their type of treatment were more likely to be LTFU compared to patients who were receiving curative chemoradiation. In our setting, palliative care services are provided at BCCOE, but most of these patients receive these services at their local district hospitals, closer to their home to reduce the burden of travelling [16]. Information sharing between facilities may be imperfect, and thus transfers of patients for palliative care may appear as LTFU in BCCOE records. It also may simply be challenging for patients, even from the Northern Province, to come for palliative care to the BCCOE due to distance. Limited studies have examined the relationship between palliative or curative care and LTFU among cancer patients. However, one study in the Netherlands showed that palliative care patients were more likely to be LTFU at the out-patient clinic compared to patients with nurse-led follow-up at home [25]. Alternative care delivery services such as home-based palliative care, home visits by frontline nurses, and calling patients who missed appointment visits should be considered in order to improve retention. This would also improve follow-up information, as some of the palliative care patients may have died and the unreported death may then appear at BCCOE as LTFU.

Another possible factor is that both physicians and patients may have negative perceptions of palliative care that contribute to LTFU. In wealthy settings, palliative care has been shown to improve patient quality of life and mood, but not clinical outcomes [26,27]. A US-based study of gynecologic oncologists found that a major barrier to initiating palliative care was the concern that patients would think the provider was “giving up” [28]. Patients on palliative care may be less likely to initiate or continue to treatment because of the sense of hopelessness to be cured. This may also mean that the palliative services offered at BCCOE do not meet the needs of these patients. We are unaware of research on provider attitudes toward palliative care in Rwanda or similar settings, but qualitative research with cancer care providers in this setting would help to shed light on this.

Missing treatment intent was also associated with late LTFU. Missing data are, of course, a consequence of LTFU – if the patient is not present, data cannot be collected. However, these data provide some insight into when during the care cascade people become LTFU. Of the late LTFU patients, 92% had cancer staging done, 95% had ECOG performance measured and 76% were supposed to have been referred to radiotherapy. Thus, most of these patients would have known their cancer stage, and the program would have been aware of their level of function. Most, in fact, had early stage cancer and ECOG below 2. It may be that a lower stage and higher function are of less concern to patients or providers than more seriously progressed disease. For these late LTFU patients who have entered the care cascade, qualitative research with both patients and providers into why patients become LTFU would be of benefit and enable the program to create interventions to retain cancer patients in care.

Given the prevalence of cervical cancer in Rwanda, and the complexity of the current cervical cancer treatment pathway that requires multi-institutional referrals, building strategies to improve community knowledge about cervical cancer, screen women for cervical cancer, connect them to care and help them navigate the health system will be imperative. At BCCOE, a training program was initiated in Burera District in 2015 to facilitate earlier diagnosis of breast cancer and has been shown to improve community health workers’ and health center nurses’ knowledge and skills in evaluating and managing breast concerns, increase the number of women visiting a health center for a breast concern, and increase the incidence of early stage breast cancer [29,30]. At the Ministry of Health’s request, this model was expanded to additional districts and to include cervical cancer screening in 2018. The model utilizes a patient navigator to ensure women with abnormal screenings complete their referral to district hospitals and/or BCCOE for additional evaluations and treatment, if necessary. However, there is currently no patient navigation program for adult patients once they enroll at BCCOE, even though the strategy has been successful in other settings in reducing LTFU and improving adherence to care [31]. Having a dedicated person to help patients navigate the numerous referrals, conduct additional patient and family education session, and proactively address barriers to care may reduce the burden associated with treatment completion and improve retention in care among patients with cervical cancer.

### Limitations

Completeness of medical records and missing data presented some challenges; however, we conducted in-depth checks of all data sources to minimize the effects of missing data on the quality of analysis and interpretation of our results. Further, “missingness’ was included as an outcome measure for all variables. Secondly, while treatment received had the strongest association with LTFU in this study, data on surgery were not available because surgery was done outside of BCCOE. However, given that chemoradiation was the main treatment option for BCCOE patients, we believe that they still have valuable importance to predict LTFU. Finally, our study was done on facility level data and may not include other potential predictors of LTFU not routinely collected in patient’s records such as wealth index and marital status. However, we believe that the information available in this study is still of great value in understanding factors associated with LTFU among cervical cancer patients.

## Conclusions

Our findings suggest that disease stage at presentation was important in early LTFU, and that treatment intent, physical function status (ECOG status), and proximity of residence to treatment center were associated with late LTFU. Understanding factors associated with LTFU at different points in treatment can help programs develop targeted interventions. Additionally, reinforcement with the clinical staff of the importance of assessing and recording patient information at every stage of treatment may alleviate some of the common problems of missing data. Decentralization of both cancer diagnostics and cancer services at different hospitals and health centers and implementing a patient navigation program may help to reduce the risk of LTFU. Lastly, we recommend that BCCOE increase follow-up for patients receiving palliative care. Strategies such as leveraging the community health workers’ system for follow-up of cervical cancer patients and support groups where patients get together and share their experiences could also be beneficial. Future qualitative research with care providers and patients in this setting to understand the influence of various aspects of the care cascade on patient and programmatic outcomes will help to provide insight and offer suggestions for program improvements if needed.
